# Characterizing Canadian funded partnered health research projects between 2011 and 2019: a retrospective analysis

**DOI:** 10.1186/s12961-023-01046-x

**Published:** 2023-09-08

**Authors:** Kathryn M. Sibley, Masood Khan, Alexie J. Touchette, Leah K. Crockett, S. Michelle Driedger, Heather L. Gainforth, Devashree Prabhu, Dawn Steliga, Olivia Tefft, Ian D. Graham

**Affiliations:** 1 Department of Community Health Sciences, University of Manitoba, Winnipeg, MB Canada; 2 Knowledge Translation, George & Fay Yee Centre for Healthcare Innovation, Winnipeg, MB Canada; 3 School of Health and Exercise Sciences, University of British Columbia, Kelowna, BC Canada; 4 International Collaboration on Repair Discoveries, University of British Columbia, Kelowna, BC Canada; 5 School of Epidemiology and Public Health, University of Ottawa, Ottawa, ON Canada; 6 Centre for Implementation Research, Ottawa Hospital Research Institute, Ottawa, ON Canada

**Keywords:** Research partnerships, Integrated knowledge translation, Community-based participatory research, Action research, Patient-oriented research

## Abstract

**Background and Aims:**

Involving research users in collaborative research approaches may increase the relevance and utility of research findings. Our primary objectives were to (i) identify and describe characteristics of Canadian federally and provincially funded health research projects that included research users and were funded between 2011 and 2019; (ii) explore changes over time; and (iii) compare characteristics between funder required and optional partnerships.

**Methods:**

Retrospective analysis. Inclusion criteria were projects that included research users. We analyzed publicly available project variables, and coded field and type of research using established classification systems. We summarized data with descriptive statistics and compared variables across three funding year blocks and partnership requirement status.

**Results:**

We identified 1153 partnered health research projects, representing 137 fields of research and 37 types of research categories. Most projects included a required partnership (80%) and fell into health and social care services research (66%). Project length and funding amount increased from average of 24.8 months and $266 248 CAD in 2011–2013 to 31.6 months and $438 766 CAD in 2017–2019. There were significantly fewer required partnerships in 2017–2019.

**Conclusions:**

Between 2011 and 2019 Canadian federally and provincially funded partnered health research reflected primarily care services research across many fields. The observed breadth suggests that partnered health research approaches are applicable in many fields of research. Additional work to support partnered research across all types of health research (especially biomedical research) is warranted. The administration of larger grants that are funded for longer time periods may address previously identified concerns among research teams engaging in partnered research but may mean that fewer teams receive funding and risk delaying responding to time-sensitive data needs for users. Our process and findings can be used as a starting point for international comparison.

**Supplementary Information:**

The online version contains supplementary material available at 10.1186/s12961-023-01046-x.

## Background

Historically, efforts to move health research into health care practices and policies have been inefficient and ineffective [1, 2]. The factors influencing research application are numerous and complex [3], and can contribute to the ‘wicked’ nature of the very problems they aim to address [4]. Collaborative research approaches that involve users of research evidence (e.g., people with lived experience of a health condition, communities, health professionals, health system decision/policy makers) in the research process may increase the relevance and utility of research findings and potential for uptake [5, 6]. Such collaborations can be guided by several approaches, including but not limited to community based participatory research (CBPR) [7], patient-oriented research (POR) [8], and integrated knowledge translation (iKT) [9]. Recently, some authors have adopted the term “health research partnerships” [10, 11] to recognize key similarities across these collaborative research approaches [12]. We note that this term may describe relationships between academic researchers and research users, research programs, and/or specific research projects. Here, we focus on the outputs of these partnerships and refer to them as “partnered health research”.

Many effects of undertaking partnered health research have been reported. These include effects on the research process and results of research, effects on relationships and the individuals involved, and effects on practices, programs, communities, policies, and systems [13, 14]. Accordingly, partnered health research has been championed by many research funding organizations around the world who seek to maximize the impact of investments on improved health and wellbeing through tailored initiatives [15–19]. In Canada, the major federal health research funding agency, the Canadian Institutes of Health Research (CIHR), has formally incorporated funding opportunities for projects that involve research users since 2004. Some project funding opportunities have specifically required a partnership between researchers and research users (such as iKT, POR, and opportunities focused on HIV and Indigenous health); in other cases a partnership may be undertaken as an optional approach in open investigator-initiated opportunities. Similarly, Canadian provincial health research funding organizations have engaged in initiatives targeting academic researchers and research users (including policy makers, practitioners and the public) to varying degrees [20].

Despite investment in and reported effects of partnered health research, the nature and critical components of partnered health research remains unclear. Existing syntheses of partnered health research are marred by limitations in both identifying partnered research and what is reported in primary studies—for example, providing only high-level case study project descriptions, only including passing identification as using a partnership approach with minimal detail, and no or minimal evaluation of outcomes [6, 10, 21]. Many syntheses are also limited in scope, focusing on specific partnership approaches (e.g. iKT [6], CBPR [22]) or fields of health research [21, 23–29]. Primary data collection and comparative analyses of partnered health research is often limited to small or specific funding networks [30, 31]. There has been no recent comprehensive study of partnered health research. The closest assessment in the Canadian context took place in a 2011 CIHR evaluation of knowledge translation funding programs between 2005 and 2010 that included an analysis of select iKT opportunities through a survey completed by 283 academic principal investigators and principal knowledge users, interviews with 50 survey respondents, and five case studies [11, 32, 33]. Key findings noted the benefits of partnered research in determining research questions, in the research process, on application in practice, and beliefs in potential research impact. However, there has been no fulsome analysis after this period, which includes the addition of a strategic funding stream for POR [34], termination of CIHR knowledge translation funding opportunities where funding was reallocated to the open investigator-initiated funding program [32], and maturation of partnered health research theory, guidance, and experiences. Exploring and understanding recent trends in partnered health research is important for identifying areas of strength, for serving as a foundation for more in-depth study, and for informing research funder and other decision making. Given international variation in health research funding approaches and structures, an analysis of one context with an established history of partnered health research, study of Canada’s experience is useful and provides relevant insight for other jurisdictions.

Our primary objectives were to: (i) identify and describe characteristics of Canadian federally and provincially funded health research projects that included research users and were funded between 2011 and 2019; (ii) explore changes over time; and (iii) compare characteristics between funder required and optional partnerships. Our secondary objective was to examine changes over time in partnered health research within federal and provincially funded initiatives, to allow for exploration of long-term national trends over time and growth of provincial initiatives.

## Methods

### Context

This study is a component of a funded set of research projects to understand current practices in Canadian partnered health research (CIHR project grant PJT # 156372). We studied the time period between 2011 and 2019 because the previous CIHR evaluation of the knowledge translation funding program included projects up to and including 2010 [32], and 2019 was the most recent year with available data at the time of data collection. Of note, Canadian research funding includes direct operating costs of research only and does not include salary costs for investigators [35].

We take an iKT approach in this work [36], defined here as a collaborative research process in which research users—those who can use the knowledge generated through research to make informed decisions [37]—are engaged in governance and conduct of the research process. Our research team includes people with lived experience of a health condition, health professionals, knowledge translation practitioners, research funding organization representatives, trainees, and academics as investigators and collaborators since project inception (prior to obtaining funding). All team members are invited to contribute to the projects to the extent that they would like to be involved and are engaged regularly through email updates and request for input and meetings (minimum of once per year but often more frequently). Involvement of team members on project components varies between and within individuals, typically spanning levels consistent with *Consult, Involve*, and/or *Collaborate* on the IAP2 Spectrum of Public Participation [38]. All team members were provided the opportunity to meet international standards for authorship of this peer-reviewed manuscript [39].

### Study design

We conducted a retrospective analysis of secondary data.

### Research project eligibility

Research projects were eligible to be included in this study if they were: (i) approved for funding by a Canadian federal or provincial health research funding organization through a peer-reviewed competition; (ii) funding approved between 2011 and 2019; and (iii) explicitly included at least one non-academic research user as principal or co-investigator on the funding application. We included CIHR and members of the National Alliance of Provincial Health Research Organizations (NAPHRO) [40] as eligible health research funding organizations because they fund all types of health research. We excluded funding organizations that fund only specific types of health research (e.g., health charities). We included projects that either were funded through opportunities that required a non-academic partner, reported involving research users on the research team or identified as using a collaborative research approach (e.g., iKT, CBPR or POR) at the time of application. We used multiple strategies to identify eligible projects. First, we searched the public CIHR Funding Decisions Database [41]. This database includes information on funded projects including (but not limited to) project title, principal investigator names, abstract, funding year, program, and funding amount. We identified eligible projects by searching specific funding opportunities and review committees that included partnership as a funding requirement along with a keyword search (see Additional file 1: Appendix 1 for a list of searched programs and keywords). We identified these programs through our collective knowledge as a research team, which included a former CIHR executive (IDG), and verified eligibility requirements through CIHR documentation. Second, we contacted CIHR for support in identifying eligible projects through a binary iKT field that was completed by applicants at the time of submission beginning in 2015 but is not available in the CIHR Funding Decisions Database. Third, we contacted the provincial NAPHRO organizations (*n* = 9) to request a list of funded projects meeting our eligibility criteria. Fourth, we searched the CIHR Funding Decisions Database for projects funded through open competitions that did not require partnership but indicated using a collaborative research approach by the presence of keywords *integrated knowledge translation*, *knowledge exchange*, *patient engagement*, and *participatory research* or were reviewed by the CIHR Knowledge Translation Research peer review committee (where we anticipated more partnerships) or the Indigenous Health peer review committee (which required partnerships). One investigator (MK) screened all projects for duplication; any uncertainties were discussed with the principal investigator (KMS) and the research team as needed.

### Data collection

Data were collected between fall 2019 and winter 2020. When a project was identified, we extracted the following data: project title, abstract, year of funding, project length (months), funding amount (CAD), funding opportunity, number of investigators named on the grant, and name of institution receiving the funding.

### Data analysis

We used the title and abstract to classify projects on two standardized classification systems. Standardized classification systems can help to understand distinctions and compare disciplines and their evolution over time [42, 43]. We used the Field of Research classifications from the Canadian Research and Development Classification [44]. This was a new classification launched by the national statistics office in 2020 as the recommended standard for all Canadian research sectors, but had not been used previously at the time of our analysis. The Field of Research contains 1663 unique codes, representing the most comprehensive categorization system available. These 1663 “subclass” codes can also be organized in a hierarchical structure that includes 168 overarching Classes, 43 Groups, and 6 Divisions. For example, the subclass code *Infectious diseases* falls within the *Clinical sciences* Class, *Clinical medicine* Group, and *Medical, health and life sciences* Division. We also used the Research Activities arm of the United Kingdom Health Research Classification System [45]. It was developed in 2004 to produce a broad overview of health research funding and has been used internationally [46, 47]. The Research Activities arm includes 48 unique Type of Research codes divided into 8 overarching code Groups. For example, the *Organization and delivery of services* type of research code falls in the *Health and social care services research* group. We chose two taxonomies to recognize the importance of using a classification specific to our Canadian context (which offered specific codes related to indigenous health and research), while also recognizing the value of established systems and potential for international comparison (which we reflected through our use of the Research Activities arm of the United Kingdom Health Research Classification System). Our approach was consistent with others who have argued that no single taxonomy can meet all needs [42].

We used the 1663 unique Field of Research subclass categories and the 48 unique Type of Research categories as primary codes. We assigned codes based on the projects’ primary aim. If a project had multiple aims, we surmised an overall goal. We chose to assign each project one most appropriate code in each classification framework. We also recorded the overarching hierarchical codes (class, group, and division) associated with each primary code. All project abstracts were coded by two reviewers independently. All reviewers first coded the same twenty randomly selected abstracts as a calibration exercise. MK coded all English abstracts with multiple second reviewers. French abstracts were independently coded by two bilingual research assistants. Projects with no abstracts were coded based on titles only if both reviewers could confidently assign a code. Coders met regularly to discuss and resolve coding discrepancies. Unresolved coding discrepancies were resolved through discussion with KMS.

Data were analyzed in SPSS (v 27). To address objective 1, we calculated descriptive statistics (mean, standard deviation, range, frequency, and proportion as appropriate) for all extracted variables as well as Field of Research and Type of Research primary codes by project. Given the high number of unique codes, we focused reporting on the ten most frequently coded categories. We also calculated the total funding investment to facilitate interpretation relative to historical data. To address objective 2, we grouped projects into three-year blocks by year of funding (2011–2013, 2014–2016, 2017–2019). We then examined relationships between project characteristics using the Pearson Chi Square test of independence for categorical variables or the Kruskal–Wallis and Mann Whitney U tests for continuous variables across funding year blocks as appropriate. Statistical significance was set at *p* < 0.05. We conducted post hoc comparisons using adjusted residuals with a Bonferonni correction for categorical variables and pairwise comparisons for continuous variables. We report the five most frequently coded categories in each funding year block. To address objective 3, we grouped projects by partnership requirement status (required or optional), calculated descriptive statistics, and compared characteristics using the Chi Square or Mann Whitney U test. For the secondary objective, we stratified projects by federal or provincial funding, and for each funding stream calculated descriptive statistics and examined relationships using the Chi Square test or Kruskal–Wallis and Mann Whitney U tests across funding year blocks.

## Results

We identified projects through all four search strategies. CIHR provided a list of projects including the binary iKT field indicated by applicants and four of the nine provincial health research funding organizations shared project data. Complete funding results for 2019 were not available at the time of data collection. We identified and included 1153 unique eligible projects.

### Objective 1: Project characteristics

Characteristics of the included projects are provided in Table 1. We identified 406 partnered health research projects funded in 2011–2013, 408 projects in 2014–2016 and 338 projects funded in 2017–2019. Most projects involved a funder required partnership (*n* = 925, 80.2%) and were funded by the federal granting agency (*n* = 851, 73.8%). The mean amount of funding per project was $337 486 CAD (SD $657 187). The total funding investment across all projects was $320 275 174 CAD. Mean project length was 27.4 months (SD 17.7). Mean number of named investigators was 9 (SD 9).

**Table 1:** Project characteristics for entire sample and by funding year block

Project characteristic	*N* (%) or Mean (SD, range)				
	Total sample (*n* = 1153)	Funding Year Block			X^2^ or H (*p* value)
		2011–2013 (*n* = 406)	2014- 2016 (*n* = 408)	2017- 2019* (*n* = 339)	
Funding type					56.7 (< 0.0001)
Federal	851 (73.8)	**349 (86**)	292 (71.5)	**210 (61.9)**	
Provincial	302 (26.2)	**57 (14)**	115 (28.5)	**129 (38.1**)	
Project funding amount (CAD) (*n* = 949)	337 487 (657 533, 5000–12 450 001)	266 248 (429 386)	336 288 (831 441)	438 766 (6 381 008)	30.1 (< 0.0001)
Project length (months) (*n* = 979)	27.4 (17.7, 1–84)	24.8 (14.6, 12–6)	27.8 (18.8, 12- 84)	31.6 (20.1, 1–84)	12.5 (0.0019)
Number of named investigators	9 (9, 1–137)	8.4 (6.2)	9.5 (9.3)	9.1 (11.2)	0.205 (0.903)
Province of funded institution (*n* = 1136)					142.6 (< 0.0001)
Ontario	347 (30.5)	**156 (38.6)**	119 (29.8)	**72 (21.6**)	
British Columbia	237 (20.9)	**62 (15.3)**	**57 (14.3)**	**118 (35.4)**	
Alberta	202 (17.8)	91 (22.5)	82 (20.5)	**29 (8.7)**	
Quebec	148 (13.0)	53 (13.1)	66 (16.5)	29 (8.7)	
Saskatchewan	99 (8.7)	10 (2.5)	42 (10.5)	47 (14.1)	
Manitoba	53 (4.7)	13 (3.2)	20 (5)	20 (6)	
Nova Scotia	43 (3.8)	19 (4.7)	12 (3)	12 (3.6)	
Other provinces/territories	7 (0.6)	0	1 (0.2)	**6 (1.8)**	

Bold denotes significant post hoc result

^*^Complete funding results were not available for 2019 at the time of data collection

We classified 1152 projects for Field of Research, which represented 185 subclass codes (range 1–57 projects per code, Additional file 2: Appendix 2). The top ten codes, representing 338 projects (29%), are reported in Table 2 with their overarching class, group, and division. The top three codes were* Infectious diseases, Healthcare safety and quality improvement*, and *Emergency care and critical care*.

**Table 2:** Top ten field of research codes

Rank	Number of projects (%)	Subclass code	Class	Group	Division
1	57 (4.9)	Infectious diseases	Clinical sciences	Clinical medicine	Medical, health and life sciences
2	53 (4.6)	Health care safety and quality improvement	Health services and systems	Health sciences	Medical, health and life sciences
3	35 (3)	Emergency care and critical care	Care	Health sciences	Medical, health and life sciences
4	34 (2.9)	Health care effectiveness and outcomes	Health services and system	Health sciences	Medical, health and life sciences
5	30 (2.6)	Mental health and wellbeing	Psychology, social and behavioural aspects	Psychology and cognitive sciences	Social sciences
6	27 (2.3)	Health equity	Public and population health	Health sciences	Medical, health and life sciences
7	26 (2.3)	Coordinated and integrated care	Care	Health sciences	Medical, health and life sciences
8	26 (2.3)	Addiction rehabilitation	Rehabilitation medicine	Health sciences	Medical, health and life sciences
9	26 (2.2)	Cardiology and circulatory sciences (including cardiovascular disease)	Cardiorespiratory medicine and hematology	Clinical medicine	Medical, health and life sciences
10	24 (2.1)	Primary health care	Care	Health sciences	Medical, health and life sciences

We classified 1143 projects for Type of Research, which represented 37 codes (range 1–479 projects per code, Additional file 3: Appendix 3). The top ten codes, representing 957 projects (83%), along with their overarching group, are reported in Table 3. The top three codes were *Policy, ethics and research governance, Organization and delivery of services,* and *Individual care needs.*

**Table 3:** Top ten type of research codes

Rank	Number of projects (%)	Type of Research code	Research activity (Group)
1	479 (41.9)	Policy, ethics and research governance	Health and social care services research
2	215 (18.8)	Organisation and delivery of services	Health and social care services research
3	68 (5.9)	Individual care needs	Management of diseases and conditions
4	61 (5.3)	Management and decision making	Management of diseases and conditions
5	31 (2.7)	Research design and methodologies	Health and social care services research
6	27 (2.4)	Resources and infrastructure (health services)	Health and social care services research
7	22 (1.9)	Psychological, social and economic factors	Aetiology
8	22 (1.9)	Primary prevention interventions to modify behaviors or promote wellbeing	Prevention of disease and conditions, and promotion of wellbeing
9	19 (1.7)	Cellular and gene therapies	Development of treatments and therapeutic interventions
10	18 (1.6)	Psychological and socioeconomic processes	Underpinning research

### Objective 2: Project characteristics over time

Characteristics by funding year block are reported in Table 1. There were significant relationships between project characteristic variables and funding year block for all variables except number of project investigators. Post hoc tests indicated numerous significant differences across funding year blocks. Of note, post hoc tests indicated significant increases in funding amount in each funding year block (all *p* < 0.021). Post hoc tests also indicated that project length was significantly greater in the 2017–2019 funding year block than the 2011–2013 block (*p* < 0.001).

Additional file 4: Appendix 4 provides the top five most frequently coded Field of Research categories for each funding year block. *Infectious diseases* and *Healthcare safety and quality improvement* were the top two in all funding year blocks. All the other top ten codes were present in the top five of at least one funding year block, except for *Coordinated and integrated care* and *Primary health care*, which were not in the top five in any funding year block. The top five Type of Research codes in each funding year block were the same as the overall top ten (Additional file 5: Appendix 5).

### Objective 3: Project characteristics by partnership type

Characteristics by partnership type are reported in Table 4. There were significant differences across funding year block, funding amount, project length, and number of named investigators between projects with funder required and optional partnership. Post hoc testing for funding year block indicated that the number of projects with required partnership was significantly higher than those with optional partnership in funding year block 2011–2013 and 2017–2019. The top Field of Research categories and Type of Research categories by partnership type are presented in Additional file 6: Appendix 6 and Additional file 7: Appendix 7.

**Table 4:** Project characteristics by partnership type

Characteristic	Projects with funder required partnership Mean (SD, range) or *N* (%)	Projects with optional partnership Mean (SD, range) or *N* (%)	*X*^2^ or *U* (*p* value)
Funder type			n/a
Federal	623 (73)	228 (27)	
Provincial	301 (100)	0	
Funding year (*n* = 1153)	*N* = 925	*N* = 228	15.3 (0.00047)
2011–13	**350 (37.8)**	**56 (24.6)**	
2014–16	320 (34.6)	88 (38.6)	
2017–19	**255 (27.6)**	**84 (36.8)**	
Funding amount (*n* = 949)	*N* = 721 220 720 (373 160, 5000–5 000 000)	*N* = 228 706, 37 (10 000–12450001, 1,087,964)	37 444.5 (< 0.0001)
Project length (months) (*n* = 979)	*N* = 751 25.7 (17.3, 1–84)	*N* = 228 38.3 (20.3, 1–84)	50 368 (< 0.0001)
Number of named investigators (*n* = 1153)	*N* = 925 7.8 (5.7, 1–62)	*N* = 228 13.7 (16, 1–137)	79 501 (< 0.0001)

Significant differences in bold, we were unable to compare provinces due to low cell counts

### Secondary objective: Changes over time in federal and provincially funded projects

Among the federally funded projects, there were significant differences in funding amount and project length across the funding year blocks (Table 5). Of note, funding amount was significantly greater and project lengths were longer in 2017–2019 relative to earlier funding year blocks (*p* < 0.0001). We were unable to conduct planned comparisons of provincially funded data due to high amounts of missing data across multiple time periods and variables. The top five Field of Research and Type of Research categories by funder type over time are presented in Additional file 8: Appendix 8 and Additional file 9: Appendix 9.

**Table 5:** Characteristics over time of federally funded projects

Project characteristic	Entire sample (*n* = 851) *N* (%) or Mean (SD, range)	2011–2013 (*n* = 349) *N* (%) or Mean (SD)	2014–2016 (*n* = 292) *N* (%) or Mean (SD)	2017–2019 (*n* = 210) *N* (%) or Mean (SD)	*X*^2^ or *H* (*p* value)
Funding amount	347 654 (659 917, 5000–12 450 001)	252 731 (347 644, 24 090–2 500 000)	349 458 (876 449, 5000–12 450 001)	502 901 (679 030, 19 995–4 996 890)	33.5 (< 0.0001)
Project length (months)	25.3 (17.4, 1–84)	22.1 (13.1, 12–60)	24.6 (18.5, 12–84)	31.6 (20.4, 1–84)	25.7 (< 0.0001)
Number of named investigators	10.4 (9.6, 1–137)	9.5 (6.1, 2–52)	11.1 (9.6, 1–71)	11.1(13.4, 1–137)	2.1 (0.342)

## Discussion

This analysis of recent trends in Canadian federally and provincially funded partnered health research is the first study to systematically classify partnered health research projects. This new knowledge about the extent of collaborative health research in Canada between 2011 and 2019 is a global first. We note three key findings: (i) a high number of Field of Research categories were identified, suggesting that Canadian partnered health research was pursued in a wide range of areas during the study time period; (ii) more than two-thirds of projects were categorized as *Health and social care services* for Type of Research; and (iii) there were shifting trends in project characteristics in the study time period, with fewer required partnerships in later years alongside longer project lengths and higher funding amounts. Our findings are most relevant for health research and funding organizations. Research organizations and funders can use this information to advance, catalyze and expand opportunities for partnered health research across a range of topics and disciplines. Our Canadian analysis provides an important template for study in other contexts that sets a foundation for international comparisons. Of note, this analysis also establishes a large partnered health research dataset that we have continued to analyze in subsequent phases of this work.

We determined that partnered health research projects funded between 2011 and 2019 in Canada had considerable breadth. No single Field of Research category applied to more than 5% of the 1152 included projects. This demonstrates that partnership approaches are versatile and widely applicable across many health research topics and that open research funding opportunities attract a range of partnered research teams. Our decision to code projects using all 1663 unique subclass categories from the Canadian Research Data Classification offered the best available precision and specificity in describing projects, and we used 11% of possible codes. Although we might have elected to restrict our coding framework, for example, to the Division of *Medical, health and life sciences* only, this would have eliminated important subclass categories, which we determined to be the most relevant primary code. For example, *Mental health and wellbeing* was the fifth most common Field of Research we coded, but it is found within the *Social sciences* Division, not *Medical, health and life sciences*. Had we restricted our codes to only the *Medical, health, and life sciences* Division, some of the most accurate categories for describing projects would have been excluded. Ultimately, using all 1663 categories was relevant, as we coded projects from all six Divisions, including *Agricultural and veterinary sciences (Indigenous food system*), *Natural sciences* (*Epigenetics and epigenomics*), and *Humanities and the arts (Religion and spirituality of Indigenous Peoples*). Application of these codes illustrate the multidisciplinary nature of Canadian partnered health research (and health research more broadly) and the critical nature of intersections between natural and social phenomena and their influence on health. As we are the first research group to apply the Canadian Research Data Classification to characterization of health research, our work also makes a methodological contribution to the science and practice of research classification. In our opinion, the extensive granularity of the new Canadian Research Data Classification System is both a strength and a limitation. More specifically, the many unique subclass categories are challenging to synthesize, although the next group up, Class, is not sufficiently specific and risks eliminating potentially relevant unique categories.

In contrast, the type of research undertaken in federally and provincially funded partnered health research projects between 2011 and 2019 in Canada was less distributed, with over 40% of projects classified as *Policy, governance or ethics research* and 66% of projects falling in the overarching *Health and social care services research* activity Group. In our sample, we categorized just 5% of projects as *Underpinning* or *Aetiogical* research. This distribution of the type of research conducted in Canadian partnered health research reflects a marked difference from overall health research patterns, where biomedical research predominates. For example, in the second half of our study period between 2016 and 2019, just 8% of all CIHR investments were identified as *Health systems and services* [48]. In contrast, investments in biomedical research ranged between 45 and 47% in this same period. Similar trends are observed globally [49]. Given the large proportion of overall health research funds directed to biomedical research, our findings support international calls to grow partnered health research in basic sciences [50] and warrant continued research to understand and support such work.

We observed that the distribution of required and optional partnerships shifted during the study period, with fewer required partnerships in the 2017–2019 block and a greater proportion of optional partnerships. This shift likely reflects a change in Canadian federal funding policies during the study period: in 2015–2016, CIHR ended several funding opportunities that required partnership and redirected funds to open funding opportunities [32]. The required partnership funding opportunities often had time and budget limits, most likely reflected in our observation that project funding amount and project length significantly increased with time (~ $266 000 CAD and 25 months in 2011–2013, ~ $439 000 CAD and 32 months in 2017–2019). This suggests that partnered research projects funded in later periods had greater time and resources. This finding is relevant to data from the 2011 CIHR knowledge translation funding program evaluation in which researchers expressed a preference for longer funding periods and increased funding amounts [32]. Although this shift could be viewed as a positive change, there is a risk of fewer teams receiving funding, and grants with longer terms being less responsive to research user needs. This finding reveals that some research teams elect to conduct partnered health research when it is not required by the funder, despite known challenges and barriers to doing so.

We can compare our findings of partnered health research funded between 2011 and 2019 in Canada with the previously published evaluation of partnered CIHR-funded projects between 2005 and 2010 [32] to explore general trends over a 15-year period. Some project characteristics seem consistent, particularly the predominance of health care and systems research foci relative to biomedical research. It is less clear whether there were meaningful shifts in funding amounts and therefore investment in partnered health research. The average funding amount in the iKT projects included in the CIHR evaluation in 2005–2010 was $107 054 CAD [32] and $337 486 CAD in the present analysis between 2011 and 2019. This three-fold increase in mean project funding may be noteworthy, although more fulsome economic analysis accounting for covariates is needed to confirm the significance of this observation.

We acknowledge the limitations of our research. First, our sample was limited to a subset of Canadian health research funders and funding opportunities. We did not include partnered research projects from other funders and charities, nor did we consider partnered research projects that did not receive peer-reviewed funding. We only received data from a minority of provincial health research funders. Second, categorizing and describing health research data is complex, and as already noted, there is no gold-standard taxonomy. We acknowledge that our decision to use a tailored combination of taxonomies affects comparison to other analyses, and that other groups might have chosen a different classification system. Third, we acknowledge that the decisions we made during coding influenced the analysis and that other teams may have made different decisions. Our approach of two coders working independently and then reaching consensus on a final code helped to mitigate bias and ensure consistency within our team. Our dollar-amount data were not adjusted for inflation. This approach is consistent with public reporting of federal funding information in Canada. We were not able to acquire a complete dataset for 2019; we were thus unable to accurately compare the number of funded partnered health research projects in this timeframe.

## Conclusions

Between 2011 and 2019, federally and provincially funded partnered health research in Canada was concentrated in health care and services but conducted across many fields of health research. This breadth of partnered health research suggests that collaborative research approaches are widely applicable across health contexts. The administration of larger grants that are funded for longer time periods may address previously identified concerns among research teams engaging in partnered research but may mean that fewer teams receive funding and risk delaying responding to time-sensitive data needs of research users. Additional study is warranted to better understand the experiences of teams engaging in partnered health research and relationships to outcomes. The low prevalence of partnership in biomedical research in Canada also warrants continued study to unpack and explore how research partnerships might play a role in basic research. Health research funding agencies in Canada have funded partnered research for over two decades. Our findings provide important insight into recent trends in Canadian partnered health research and can be used as a foundation for continued study and international comparison. Given the multidisciplinary nature of the partnered research we characterized, it will also be important to expand future studies of partnered research to the social and natural sciences.

### Supplementary Information

**Additional file 1: Appendix 1. **Searched programs and keywords.

**Additional file 2: Appendix 2. **Field of Research codes (*N* = 1152).

**Additional file 3: Appendix 3. **Type of Research codes (*N* = 1143).

**Additional file 4: Appendix 4. **Top five Field of Research codes by funding year block.

**Additional file 5: Appendix 5. **Top five Type of Research codes by funding year block.

**Additional file 6: Appendix 6. **Top five Field of Research codes by partnership type.

**Additional file 7: Appendix 7. **Top five Type of Research codes by partnership type.

**Additional file 8: Appendix 8. **Top five Field of Research codes over time by funder type.

**Additional file 9: Appendix 9. **Top five Type of Research codes over time by funder type.

## Data Availability

The datasets used and analyzed during the current study are available from the corresponding author on reasonable request.

## Abbreviations

CBPR: Community based participatory research
POR: Patient-oriented research
iKT: Integrated knowledge translation
CIHR: Canadian Institutes of Health Research
NAPHRO: National Alliance of Provincial Health Research Organizations

## References

[1] Balas E, Boren S, Bemmel J, McCray AT (2000). Managing clinical knowledge for health care improvement. Yearbook of medical informatics: patient centered systems.

[2] Kothari A, Rycroft-Malone J, McCutcheon C, Graham ID, Graham ID, Rycroft-Malone J, Kothari A, McCutcheon C (2022). Introduction. Research co-production in healthcare.

[3] Damschroder L, Reardon CM, Widerquist MAO (2022). The updated consolidated framework for implementation research based on user feedback. Implement Sci.

[4] Haynes A, Garvey K, Davidson S, Milat A (2020). What can policy-makers get out of systems thinking? Policy partners' experiences of a systems-focused research collaboration in preventive health. Int J Health Policy Manag.

[5] Jagosh J, Macaulay AC, Pluye P, Salsberg J, Bush PL, Henderson J (2012). Uncovering the benefits of participatory research: implications of a realist review for health research and practice. Milbank Q.

[6] Gagliardi AR, Berta W, Kothari A, Boyko J, Urquhart R (2016). Integrated knowledge translation (IKT) in health care: a scoping review. Implement Sci.

[7] Israel BA, Schulz AJ, Parker EA, Becker AB (2001). Community-based participatory research: policy recommendations for promoting a partnership approach in health research. Educ Health (Abingdon).

[8] Strategy for Patient-Oriented Research - Patient Engagement Framework: Canadian Institutes of Health Research; 2019. https://cihr-irsc.gc.ca/e/48413.html.

[9] CE Handbook - Chapter 6: Research Priority Setting and Integrated Knowledge Translation (Focus Area 3): Canadian Institutes of Health Research; 2010; https://cihr-irsc.gc.ca/e/42211.html.

[10] Hoekstra F, Mrklas KJ, Khan M, McKay RC, Vis-Dunbar M, Sibley KM (2020). A review of reviews on principles, strategies, outcomes and impacts of research partnerships approaches: a first step in synthesising the research partnership literature. Health Res Policy Syst.

[11] Sibbald SL, Tetroe J, Graham ID (2014). Research funder required research partnerships: a qualitative inquiry. Implement Sci.

[12] Nguyen T, Graham ID, Mrklas KJ, Bowen S, Cargo M, Estabrooks CA (2020). How does integrated knowledge translation (IKT) compare to other collaborative research approaches to generating and translating knowledge? Learning from experts in the field. Health Res Policy Syst.

[13] Sibley KM, Hoekstra F, Kothari A, Mrklas KJ, Graham ID, Rycroft-Malone J, Kothari A, McCutcheon C (2022). Effects, facilitators, and barriers of research coproduction reported in peer-reviewed literature. Research coproduction in healthcare.

[14] Mrklas KJMS, Khan M, Shergill S, Boyd JM, Nowell L, Pfadenhauer LM, Paul K, Goertzen A, Swain L, Sibley KM, Vis-Dunbar M, Hill MD, Raffin-Bouchal S, Tonelli M, Graham ID (2022). How are health research partnerships assessed? A systematic review of outcomes, impacts, terminology and the use of theories, models and frameworks. Health Res Policy Syst..

[15] Graham ID, Tetroe J (2007). How to translate health research knowledge into effective healthcare action. Healthc Q.

[16] Kerner JF (2006). Knowledge translation versus knowledge integration: a "funder's" perspective. J Contin Educ Health Prof.

[17] PCORI: Patient-Centered Outcomes Research Institute; 2022; https://www.pcori.org/.

[18] Collaborating in applied health research: National Institute for Health and Care Research; 2022; https://www.nihr.ac.uk/explore-nihr/support/collaborating-in-applied-health-research.htm.

[19] Partnership Centres for Better Health: National Health and Medical Research Council- Australian Government; 2021; https://www.nhmrc.gov.au/research-policy/research-translation-and-impact/partnership-centres-better-health.

[20] Holmes B, Scarrow G, Schellenberg M (2012). Translating evidence into practice: the role of health research funders. Implement Sci.

[21] Camden C, Shikako-Thomas K, Nguyen T, Graham E, Thomas A, Sprung J (2015). Engaging stakeholders in rehabilitation research: a scoping review of strategies used in partnerships and evaluation of impacts. Disabil Rehabil.

[22] Dickson E, Magarati M, Boursaw B, Oetzel J, Devia C, Ortiz K (2020). Characteristics and practices within research partnerships for health and social equity. Nurs Res.

[23] Ehde DM, Wegener ST, Williams RM, Ephraim PL, Stevenson JE, Isenberg PJ (2013). Developing, testing, and sustaining rehabilitation interventions via participatory action research. Arch Phys Med Rehabil.

[24] Haijes HA, van Thiel GJMW (2016). Participatory methods in pediatric participatory research: a systematic review. Pediatr Res.

[25] Hubbard G, Kidd L, Donaghy E (2008). Involving people affected by cancer in research: a review of literature. Eur J Cancer Care.

[26] Miller J, Knott VE, Wilson C, Roder D (2012). A review of community engagement in cancer control studies among Indigenous people of Australia, New Zealand, Canada and the USA. Eur J Cancer Care.

[27] Orlowski SK, Lawn S, Venning A, Winsall M, Jones GM, Wyld K (2015). Participatory research as one piece of the puzzle: a systematic review of consumer involvement in design of technology-based youth mental health and well-being interventions. JMIR Hum Factors.

[28] Stacciarini JM, Shattell MM, Coady M, Wiens B (2011). Review: community-based participatory research approach to address mental health in minority populations. Community Ment Health J.

[29] Vaughn LM, Wagner E, Jacquez F (2013). A review of community-based participatory research in child health. MCN Am J Matern Child Nurs.

[30] Roberge-Dao J, Yardley B, Menon A, Halle MC, Maman J, Ahmed S (2019). A mixed-methods approach to understanding partnership experiences and outcomes of projects from an integrated knowledge translation funding model in rehabilitation. BMC Health Serv Res.

[31] Kislov R, Wilson PM, Knowles S, Boaden R (2018). Learning from the emergence of NIHR Collaborations for Leadership in Applied Health Research and Care (CLAHRCs): a systematic review of evaluations. Implement Sci.

[32] McLean R, Tucker J. Evaluation of CIHR's Knowledge Translation Funding Program. Canadian Institutes of Health Research; 2013.

[33] Sibbald SL, Kang H, Graham ID (2019). Collaborative health research partnerships: a survey of researcher and knowledge-user attitudes and perceptions. Health Res Policy Syst.

[34] Canadian Institutes of Health Research. Canada's Strategy for Patient-oriented Research: Improving Health Outcomes Through Evidence-informed Care: Canadian Institutes of Health Research; 2013.

[35] Tri-agency Guide on Financial Administration: Natural Sciences and Engineering Research Council of Canada: Tri-agency financial administration, Government of Canada; 2022; https://www.nserc-crsng.gc.ca/InterAgency-Interorganismes/TAFA-AFTO/guide-guide_eng.asp#9.

[36] Kothari A, McCutcheon C, Graham ID (2017). Defining integrated knowledge translation and moving forward: a response to recent commentaries. Int J Health Policy Manag.

[37] Canadian Institutes of Health Research. Glossary of funding-related terms [updated February 16, 2021; https://cihr-irsc.gc.ca/e/34190.html. Accessed 5 Jul 2021.

[38] IAP2 Spectrum of Public Participation. 42 International Association for Public Participation IAP2 Spectrum of Public Participation Denver: International Association for Public Participation; 2018

[39] International Committee of Medical Journal Editors. Defining the role of authors and contributors: Defining the Role of Authors and Contributors: [cited 2021 July 5].

[40] The National Alliance of Provincial Health Research Organizations https://www.naphro.ca/.

[41] CIHR. Funding Decisions Database. https://webapps.cihr-irsc.gc.ca/decisions/p/main.html?lang=en#sort=namesort%20asc&start=0&rows=20.

[42] Alavi M, Leidner DE (2001). Review: knowledge management and knowledge management systems: conceptual foundations and research issues. MIS Q.

[43] Legendre A. Canadian Research and Development Classification (CRDC) 2019. https://www.isko.org/cyclo/crdc.

[44] Canadian Research and Development Classification (CRDC) 2020 Version 1.0. 2020. https://www.statcan.gc.ca/eng/subjects/standard/crdc/2020v1/index.

[45] UKCRC Health Research Classification System. UK Clinical Research Collaboration. 2018. http://hrcsonline.net/wp-content/uploads/2018/01/HRCS_Main_Handbook_v2_Feb2018.pdf.

[46] Nicolau I, Ling D, Tian L, Lienhardt C, Pai M (2012). Research questions and priorities for tuberculosis: a survey of published systematic reviews and meta-analyses. PLoS ONE.

[47] Clyne B, Boland F, Murphy N, Murphy E, Moriarty F, Barry A (2020). Quality, scope and reporting standards of randomised controlled trials in Irish Health Research: an observational study. Trials.

[48] CIHR in Numbers: Canadian Institutes of Health Research; https://cihr-irsc.gc.ca/e/50218.html.

[49] UK Health Research Analysis 2018: UK Clinical Research Collaboration; 2020; https://hrcsonline.net/wp-content/uploads/2020/01/UK-Health-Research-Analysis-2018-for-web-v1-28Jan2020.pdf.

[50] Dobbs T, Whitaker I. Patient and public involvement in basic science research–are we doing enough. The BMJ opinion (blog). 2016;11.

