# Supplementary material for: Characterizing Canadian funded partnered health research projects between 2011 and 2019: a retrospective analysis
Source: Health Res Policy Syst. 2023 Sep 8;21:92. doi: 10.1186/s12961-023-01046-x (PMC10492355; doi:10.1186/s12961-023-01046-x)
Supplement: Supplementary file 1 — Additional file 1: Appendix 1. Searched programs and keywords. [file 12961_2023_1046_MOESM1_ESM.pdf]

Appendix 1: Searched programs and keywords

**CIHR program name or provincial funder**	**Partnership requirement**	**Number of grants**	**Maximum budget ($)**	**Maximum term (months)**
Knowledge Synthesis Grant	Mandatory	157	100000	112
Indigenous Health Research Peer Review Committee grants	Mandatory	101	Varied (range= 25000-2505376)	Varied (range= 12-60)
Patient-Oriented Research Collaboration Grants	Mandatory	90	100000	12
Partnerships for Health System Improvement	Mandatory	87	400000	36
Knowledge Translation Supplement	Mandatory	68	100000	12
Search term "participatory research"	Optional	67	Varied (range=5000-2500000)	Varied (range 1-84)
Knowledge Translation Research Peer Review Committee grants	Optional	55	Varied (range= 82348-2096101)	Varied (range= 12-60)
Operating Grant: Knowledge to Action	Mandatory	49	200000	24
Self-identifying integrated knowledge translation projects (*list provided by CIHR*)	Optional	36	Varied (range= 75000-1051875)	Varied (range= 12-60)
Catalyst Grant: HIV/AIDS Community Based Research Program	Mandatory	24	40000	12
Search term "integrated knowledge translation"	Optional	24	Varied (range= 99333-3972033)	Varied (range= 12-84)
Search term "patient engagement"	Optional	24	Varied (range= 49948-12450001)	Varied (range 12-84)
Search term "knowledge exchange"	Optional	22	Varied (range= 10,000-3780858)	Varied (range= 12-84)
Operating Grant - HIV/AIDS CBR Program	Mandatory	14	450000	36
Patient-Oriented Research Collaboration Grants -New/Early Career Investigators	Mandatory	10	40000	12
Other formal integrated knowledge translation grants (*list provided by CIHR*)	Mandatory	9	Varied (range=25000-180000)	Varied between 6-12
Evidence on Tap - Expedited Knowledge Synthesis	Mandatory	8	100000	12
Operating Grant - Priority Announcement: Knowledge Translation	Mandatory	6	100000	12

Projects funded by Alberta Innovates	Unknown	111	Unknown	Varied (range= 12-60)
Projects funded by MSFHR	Mandatory	93	Unknown	Unknown
Projects funded by SHRF	Mandatory	78	Varied (range= 25000-180000)	Unknown
Project funded by Research MB	Unknown	20	Varied (range= 20000-5000000)	Varied (range= 12-60)
