# Supplementary material for: Characterizing Canadian funded partnered health research projects between 2011 and 2019: a retrospective analysis
Source: Health Res Policy Syst. 2023 Sep 8;21:92. doi: 10.1186/s12961-023-01046-x (PMC10492355; doi:10.1186/s12961-023-01046-x)
Supplement: Supplementary file 2 — Additional file 2: Appendix 2. Field of Research codes (N = 1152). [file 12961_2023_1046_MOESM2_ESM.pdf]

Appendix 2. Field of Research codes (N=1152)

**Code (Sub-class > Class > Group > Division)**	N (%)
RDF3020213 Infectious diseases > RDF30202 Clinical sciences > RDF302 Clinical medicine > RDF30 Medical, health and life sciences	57 (4.9)
RDF3030407 Health care safety and quality improvement > RDF30304 Health services and systems > RDF303 Health sciences > RDF30 Medical, health and life sciences	53 (4.6)
RDF3030506 Emergency care and critical care > RDF30305 Care > RDF303 Health sciences > RDF30 Medical, health and life sciences	35 (3)
RDF3030406 Health care effectiveness and outcomes > RDF30304 Health services and systems > RDF303 Health sciences > RDF30 Medical, health and life sciences	34 (2.9)
RDF5010121 Mental health and wellbeing > RDF50101 Psychology, social and behavioural aspects > RDF501 Psychology and cognitive sciences > RDF50 Social sciences	30 (2.6)
RDF3030214 Health equity > RDF30302 Public and population health > RDF303 Health sciences > RDF30 Medical, health and life sciences	27 (2.3)
RDF3030509 Coordinated and integrated care > RDF30305 Care > RDF303 Health sciences > RDF30 Medical, health and life sciences	26 (2.3)
RDF3030306 Addiction rehabilitation > RDF30303 Rehabilitation medicine > RDF303 Health sciences > RDF30 Medical, health and life sciences	26 (2.3)
RDF3020101 Cardiology and circulatory sciences (including cardiovascular disease) > RDF30201 Cardiorespiratory medicine and hematology > RDF302 Clinical medicine > RDF30 Medical, health and life sciences	26 (2.3)
RDF3030502 Primary health care > RDF30305 Care > RDF303 Health sciences > RDF30 Medical, health and life sciences	24 (2.1)
RDF3030204 Prevention, treatment and support of youth health > RDF30302 Public and population health > RDF303 Health sciences > RDF30 Medical, health and life sciences	20 (1.7)
RDF3030212 Health promotion and disease prevention > RDF30302 Public and population health > RDF303 Health sciences > RDF30 Medical, health and life sciences	19 (1.6)
RDF3030513 Maternal and newborn care > RDF30305 Care > RDF303 Health sciences > RDF30 Medical, health and life sciences	17 (1.5)
RDF3010204 Cancer diagnosis > RDF30102 Cancer > RDF301 Basic medicine and life sciences > RDF30 Medical, health and life sciences	17 (1.5)
RDF3030514 Health care access, privilege or marginalization > RDF30305 Care > RDF303 Health sciences > RDF30 Medical, health and life sciences	17 (1.5)
RDF3030512 Palliation and end-of-life care > RDF30305 Care > RDF303 Health sciences > RDF30 Medical, health and life sciences	16 (1.4)
RDF3020103 Respiratory diseases > RDF30201 Cardiorespiratory medicine and hematology > RDF302 Clinical medicine > RDF30 Medical, health and life sciences	16 (1.4)

RDF3030201 Indigenous health > RDF30302 Public and population health > RDF303 Health sciences > RDF30 Medical, health and life sciences	16 (1.3)
RDF3030504 Aged health care services (except nursing) > RDF30305 Care > RDF303 Health sciences > RDF30 Medical, health and life sciences	15 (1.3)
RDF5040107 Sociological methodology and research methods > RDF50401 Sociology > RDF504 Sociology and related studies > RDF50 Social sciences	15 (1.3)
RDF3030216 Gender and health relationship > RDF30302 Public and population health > RDF303 Health sciences > RDF30 Medical, health and life sciences	14 (1.2)
RDF5060213 Health policy > RDF50602 Policy and policy administration > RDF506 Political science and policy administration > RDF50 Social sciences	14 (1.2)
RDF3010704 Exercise physiology > RDF30107 Musculoskeletal health and human movement > RDF301 Basic medicine and life sciences > RDF30 Medical, health and life sciences	14 (1.2)
RDF3020209 Clinical sciences on obesity > RDF30202 Clinical sciences > RDF302 Clinical medicine > RDF30 Medical, health and life sciences	13 (1.1)
RDF3020215 Nephrology > RDF30202 Clinical sciences > RDF302 Clinical medicine > RDF30 Medical, health and life sciences	13 (1.1)
RDF3020223 Psychiatry (including psychotherapy) > RDF30202 Clinical sciences > RDF302 Clinical medicine > RDF30 Medical, health and life sciences	13 (1.1)
RDF3030205 Psychosocial, sociocultural and behavioural determinants of health > RDF30302 Public and population health > RDF303 Health sciences > RDF30 Medical, health and life sciences	13 (1.1)
RDF3020208 Clinical sciences on diabetes > RDF30202 Clinical sciences > RDF302 Clinical medicine > RDF30 Medical, health and life sciences	12 (1)
RDF3030409 Patient and citizen engagement research > RDF30304 Health services and systems > RDF303 Health sciences > RDF30 Medical, health and life sciences	11 (1)
RDF3030511 Patient-centered care > RDF30305 Care > RDF303 Health sciences > RDF30 Medical, health and life sciences	10 (0.9)
RDF3030410 Electronic health (e-Health) > RDF30304 Health services and systems > RDF303 Health sciences > RDF30 Medical, health and life sciences	10 (0.9)
RDF3020507 Fertility and maternal health > RDF30205 Pediatrics and reproductive medicine > RDF302 Clinical medicine > RDF30 Medical, health and life sciences	10 (0.9)
RDF3020204 Sexually transmitted diseases and infections > RDF30202 Clinical sciences > RDF302 Clinical medicine > RDF30 Medical, health and life sciences	10 (0.9)
RDF3010419 Neurological disorders (except neuromuscular diseases) > RDF30104 Neurosciences, medical and physiological and health aspects > RDF301 Basic medicine and life sciences > RDF30 Medical, health and life sciences	10 (0.9)
RDF3030501 Family care > RDF30305 Care > RDF303 Health sciences > RDF30 Medical, health and life sciences	10 (0.9)

RDF3030401 Health and community services > RDF30304 Health services and systems > RDF303 Health sciences > RDF30 Medical, health and life sciences	10 (0.9)
RDF3020219 Orthopedics > RDF30202 Clinical sciences > RDF302 Clinical medicine > RDF30 Medical, health and life sciences	10 (0.9)
RDF3030403 Health information systems (including surveillance) > RDF30304 Health services and systems > RDF303 Health sciences > RDF30 Medical, health and life sciences	10 (0.9)
RDF3020303 Traditional Indigenous medicine and treatments > RDF30203 Complementary and alternative medicine > RDF302 Clinical medicine > RDF30 Medical, health and life sciences	10 (0.9)
RDF3030503 Residential client care > RDF30305 Care > RDF303 Health sciences > RDF30 Medical, health and life sciences	10 (0.9)
RDF3020210 Gastroenterology > RDF30202 Clinical sciences > RDF302 Clinical medicine > RDF30 Medical, health and life sciences	10 (0.9)
RDF3030411 Mobile health (mHealth) > RDF30304 Health services and systems > RDF303 Health sciences > RDF30 Medical, health and life sciences	9 (0.8)
RDF3030206 Environmental determinants of health (including environment-gene interactions) > RDF30302 Public and population health > RDF303 Health sciences > RDF30 Medical, health and life sciences	9 (0.8)
RDF3010203 Cancer drug development and therapeutics > RDF30102 Cancer > RDF301 Basic medicine and life sciences > RDF30 Medical, health and life sciences	9 (0.8)
RDF3020225 Surgery > RDF30202 Clinical sciences > RDF302 Clinical medicine > RDF30 Medical, health and life sciences	9 (0.8)
RDF3010408 Sensory systems, pain > RDF30104 Neurosciences, medical and physiological and health aspects > RDF301 Basic medicine and life sciences > RDF30 Medical, health and life sciences	9 (0.8)
RDF3030408 Knowledge translation and implementation science in health > RDF30304 Health services and systems > RDF303 Health sciences > RDF30 Medical, health and life sciences	9 (0.8)
RDF3030207 Ethical, legal, and social issues in health, health systems and health research > RDF30302 Public and population health > RDF303 Health sciences > RDF30 Medical, health and life sciences	9 (0.8)
RDF3020226 Clinical oncology > RDF30202 Clinical sciences > RDF302 Clinical medicine > RDF30 Medical, health and life sciences	9 (0.8)
RDF5030209 Medicine, nursing and health curriculum, pedagogy and didactics > RDF50302 Curriculum, pedagogy and didactics > RDF503 Education > RDF50 Social sciences	8 (0.7)
RDF3030305 Mental health rehabilitation > RDF30303 Rehabilitation medicine > RDF303 Health sciences > RDF30 Medical, health and life sciences	7 (0.6)
RDF3010299 Cancer, n.e.c. > RDF30102 Cancer > RDF301 Basic medicine and life sciences > RDF30 Medical, health and life sciences	7 (0.6)
RDF3020104 Cerebrovascular sciences (including stroke) > RDF30201 Cardiorespiratory medicine and hematology > RDF302 Clinical medicine > RDF30 Medical, health and life sciences	7 (0.6)
RDF3010706 Balance, gait, and locomotion > RDF30107 Musculoskeletal health and human movement > RDF301 Basic medicine and life sciences > RDF30 Medical, health and life sciences	6 (0.5)

RDF3010201 Cancer progression and metastasis > RDF30102 Cancer > RDF301 Basic medicine and life sciences > RDF30 Medical, health and life sciences	6 (0.5)
RDF5010109 Clinical psychology > RDF50101 Psychology, social and behavioural aspects > RDF501 Psychology and cognitive sciences > RDF50 Social sciences	6 (0.5)
RDF3040104 Regenerative medicine (including stem cells and tissue engineering) > RDF304 Medical biotechnology > RDF30 Medical, health and life sciences > RDF30 Medical, health and life sciences	6 (0.5)
RDF3030213 Food security > RDF30302 Public and population health > RDF303 Health sciences > RDF30 Medical, health and life sciences	6 (0.5)
RDF5020110 Economics of health care > RDF50201 Economics > RDF502 Economics and business administration > RDF50 Social sciences	6 (0.5)
RDF3010414 Neurodegeneration > RDF30104 Neurosciences, medical and physiological and health aspects > RDF301 Basic medicine and life sciences > RDF30 Medical, health and life sciences	6 (0.5)
RDF3020229 Rare diseases > RDF30202 Clinical sciences > RDF302 Clinical medicine > RDF30 Medical, health and life sciences	6 (0.5)
RDF3010307 Vaccines > RDF30103 Medical microbiology > RDF301 Basic medicine and life sciences > RDF30 Medical, health and life sciences	6 (0.5)
RDF1010302 Biostatistical methods > RDF10103 Statistics > RDF101 Mathematics and statistics > RDF10 Natural sciences	5 (0.4)
RDF3030515 Home health care > RDF30305 Care > RDF303 Health sciences > RDF30 Medical, health and life sciences	5 (0.4)
RDF3030405 Community child health > RDF30304 Health services and systems > RDF303 Health sciences > RDF30 Medical, health and life sciences	5 (0.4)
RDF3010208 Solid cancer tumours > RDF30102 Cancer > RDF301 Basic medicine and life sciences > RDF30 Medical, health and life sciences	5 (0.4)
RDF3010501 Basic pharmacology > RDF30105 Pharmacology and pharmaceutical sciences (except clinical aspects) > RDF301 Basic medicine and life sciences > RDF30 Medical, health and life sciences	5 (0.4)
RDF3030203 Environmental, developmental and social factors of youth health> RDF30302 Public and population health > RDF303 Health sciences> RDF30 Medical, health and life sciences	5 (0.4)
RDF3020502 Obstetrics and gynecology > RDF30205 Pediatrics and reproductive medicine > RDF302 Clinical medicine > RDF30 Medical, health and life sciences	5 (0.4)
RDF3020503 Pediatrics > RDF30205 Pediatrics and reproductive medicine > RDF302 Clinical medicine > RDF30 Medical, health and life sciences	5 (0.4)
RDF3010415 Neurodevelopment > RDF30104 Neurosciences, medical and physiological and health aspects > RDF301 Basic medicine and life sciences > RDF30 Medical, health and life sciences	4 (0.3)
RDF2070104 Medical devices > RDF20701 Medical and biomedical engineering > RDF207 Medical and biomedical engineering > RDF20-21 Engineering and technology	4 (0.3)

RDF3020102 Hematology > RDF30201 Cardiorespiratory medicine and hematology > RDF302 Clinical medicine > RDF30 Medical, health and life sciences	4 (0.3)
RDF3010207 Cancer molecular targets > RDF30102 Cancer > RDF301 Basic medicine and life sciences > RDF30 Medical, health and life sciences	4 (0.3)
RDF3030210 Environmental and occupational health and safety > RDF30302 Public and population health > RDF303 Health sciences > RDF30 Medical, health and life sciences	4 (0.3)
RDF3020399 Complementary and alternative medicine, n.e.c. > RDF30203 Complementary and alternative medicine > RDF302 Clinical medicine > RDF30 Medical, health and life sciences	4 (0.3)
RDF3010504 Drug discovery, design and delivery > RDF30105 Pharmacology and pharmaceutical sciences (except clinical aspects) > RDF301 Basic medicine and life sciences > RDF30 Medical, health and life sciences	4 (0.3)
RDF3020499 Dentistry and oral health, n.e.c. > RDF30204 Dentistry and oral health > RDF302 Clinical medicine > RDF30 Medical, health and life sciences	4 (0.3)
RDF3030505 Services for persons with disabilities > RDF30305 Care > RDF303 Health sciences > RDF30 Medical, health and life sciences	4 (0.3)
RDF3020501 Fetal development and medicine > RDF30205 Pediatrics and reproductive medicine > RDF302 Clinical medicine > RDF30 Medical, health and life sciences	4 (0.3)
RDF3030516 Traumatology > RDF30305 Care > RDF303 Health sciences > RDF30 Medical, health and life sciences	4 (0.3)
RDF3010404 Neuromuscular diseases > RDF30104 Neurosciences, medical and physiological and health aspects > RDF301 Basic medicine and life sciences > RDF30 Medical, health and life sciences	4 (0.3)
RDF3030218 Health and gender based violence > RDF30302 Public and population health > RDF303 Health sciences > RDF30 Medical, health and life sciences	4 (0.3)
RDF3010109 Transplantation immunology > RDF30101 Immunology > RDF301 Basic medicine and life sciences > RDF30 Medical, health and life sciences	4 (0.3)
RDF3030202 Population health interventions > RDF30302 Public and population health > RDF303 Health sciences > RDF30 Medical, health and life sciences	4 (0.3)
RDF2020105 Assistive technologies > RDF20201 Human factor engineering > RDF202 Industrial, systems and processes engineering > RDF20-21 Engineering and technology	3 (0.3)
RDF3040102 Medical biotechnology diagnostics (including biosensors) > RDF30401 Medical biotechnology > RDF304 Medical biotechnology > RDF30 Medical, health and life sciences	3 (0.3)
RDF3020228 Ophthalmology > RDF30202 Clinical sciences > RDF302 Clinical medicine > RDF30 Medical, health and life sciences	3 (0.3)
RDF3030101 Clinical nutrition > RDF30301 Human nutrition and dietetics > RDF303 Health sciences > RDF30 Medical, health and life sciences	3 (0.3)
RDF3010701 Bone, skin and cartilage science > RDF30107 Musculoskeletal health and human movement > RDF301 Basic medicine and life sciences > RDF30 Medical, health and life sciences	3 (0.3)

RDF3030103 Dietetics and nutrigenomics > RDF30301 Human nutrition and dietetics > RDF303 Health sciences > RDF30 Medical, health and life sciences	3 (0.3)
RDF3020407 Pedodontics > RDF30204 Dentistry and oral health > RDF302 Clinical medicine > RDF30 Medical, health and life sciences	3 (0.3)
RDF3040101 Gene and molecular therapy > RDF30401 Medical biotechnology > RDF304 Medical biotechnology > RDF30 Medical, health and life sciences	3 (0.3)
RDF3020218 Radiology, nuclear medicine and medical imaging > RDF30202 Clinical sciences > RDF302 Clinical medicine > RDF30 Medical, health and life sciences	3 (0.3)
RDF3030209 Social and biological determinants of aging > RDF30302 Public and population health > RDF303 Health sciences > RDF30 Medical, health and life sciences	3 (0.3)
RDF3010205 Cancer genetics > RDF30102 Cancer > RDF301 Basic medicine and life sciences > RDF30 Medical, health and life sciences	3 (0.3)
RDF3010209 Pediatric cancer > RDF30102 Cancer > RDF301 Basic medicine and life sciences > RDF30 Medical, health and life sciences	3 (0.3)
RDF3030402 Health care administration > RDF30304 Health services and systems > RDF303 Health sciences > RDF30 Medical, health and life sciences	3 (0.3)
RDF3020212 Geriatrics and gerontology > RDF30202 Clinical sciences > RDF302 Clinical medicine > RDF30 Medical, health and life sciences	3 (0.3)
RDF5010209 Sleep (including sleep behaviour and development) > RDF50102 Cognitive sciences > RDF501 Psychology and cognitive sciences > RDF50 Social sciences	2 (0.2)
RDF3020227 Neurology > RDF30202 Clinical sciences > RDF302 Clinical medicine > RDF30 Medical, health and life sciences	2 (0.2)
RDF5010105 Forensic psychology > RDF50101 Psychology, social and behavioural aspects > RDF501 Psychology and cognitive sciences > RDF50 Social sciences	2 (0.2)
RDF3020217 Clinical and translational cardiovascular sciences > RDF30202 Clinical sciences > RDF302 Clinical medicine > RDF30 Medical, health and life sciences	2 (0.2)
RDF3020207 Human metabolism and diseases (except diabetes and obesity) > RDF30202 Clinical sciences > RDF302 Clinical medicine > RDF30 Medical, health and life sciences	2 (0.2)
RDF4010109 Indigenous food system > RDF40101 Agriculture, land and farm management > RDF401 Agriculture, forestry, and fisheries > RDF40 Agricultural and veterinary sciences	2 (0.2)
RDF3010502 Pharmacogenomics > RDF30105 Pharmacology and pharmaceutical sciences (except clinical aspects) > RDF301 Basic medicine and life sciences > RDF30 Medical, health and life sciences	2 (0.2)
RDF1060605 Epigenetics and epigenomics > RDF10606 Genetics > RDF106 Biological sciences > RDF10 Natural sciences	2 (0.2)
RDF5090402 Counselling, welfare and community services > RDF50904 Social work > RDF509 Other social sciences > RDF50 Social sciences	2 (0.2)

RDF5010112 Clinical child psychology > RDF50101 Psychology, social and behavioural aspects > RDF501 Psychology and cognitive sciences > RDF50 Social sciences	2 (0.2)
RDF3010505 Psychopharmacology > RDF30105 Pharmacology and pharmaceutical sciences (except clinical aspects) > RDF301 Basic medicine and life sciences > RDF30 Medical, health and life sciences	2 (0.2)
RDF5080106 Social media studies > RDF50801 Communication and media studies > RDF508 Media and communications > RDF50 Social sciences	2 (0.2)
RDF3020231 Translational and clinical pharmacology, and therapeutics > RDF30202 Clinical sciences > RDF302 Clinical medicine > RDF30 Medical, health and life sciences	2 (0.2)
RDF5020203 Occupational health, safety and wellness management > RDF50202 Industrial relations and work relations > RDF502 Economics and business administration > RDF50 Social sciences	2 (0.2)
RDF3010705 Sensorimotor control > RDF30107 Musculoskeletal health and human movement > RDF301 Basic medicine and life sciences > RDF30 Medical, health and life sciences	2 (0.2)
RDF5010101 Psychology of addiction > RDF50101 Psychology, social and behavioural aspects > RDF501 Psychology and cognitive sciences > RDF50 Social sciences	2 (0.2)
RDF1060302 Community ecology (except invasive species ecology) > RDF10603 Ecology (except applications) > RDF106 Biological sciences > RDF10 Natural sciences	2 (0.2)
RDF5010107 Sexual identity, sexual attraction and sexual behaviour > RDF50101 Psychology, social and behavioural aspects > RDF501 Psychology and cognitive sciences > RDF50 Social sciences	2 (0.2)
RDF3020232 Clinical pharmacy and pharmacy practice > RDF30202 Clinical sciences > RDF302 Clinical medicine > RDF30 Medical, health and life sciences	2 (0.2)
RDF2040204 Automotive safety engineering > RDF20402 Automotive engineering > RDF204 Mechanical engineering > RDF20-21 Engineering and technology	2 (0.2)
RDF3030302 Physical therapy > RDF30303 Rehabilitation medicine > RDF303 Health sciences > RDF30 Medical, health and life sciences	2 (0.2)
RDF3030304 Speech pathology > RDF30303 Rehabilitation medicine > RDF303 Health sciences > RDF30 Medical, health and life sciences	2 (0.2)
RDF3020224 Rheumatology > RDF30202 Clinical sciences > RDF302 Clinical medicine > RDF30 Medical, health and life sciences	2 (0.2)
RDF3030399 Rehabilitation medicine, n.e.c. > RDF30303 Rehabilitation medicine > RDF303 Health sciences > RDF30 Medical, health and life sciences	2 (0.2)
RDF3030404 Health counselling > RDF30304 Health services and systems > RDF303 Health sciences > RDF30 Medical, health and life sciences	2 (0.2)
RDF3030199 Human nutrition and dietetics, n.e.c. > RDF30301 Human nutrition and dietetics > RDF303 Health sciences > RDF30 Medical, health and life sciences	2 (0.2)

RDF1040502 Molecular modelling and design > RDF10405 Biomolecular and medicinal chemistry > RDF104 Chemical sciences > RDF10 Natural sciences	1 (0.1)
RDF5010208 Cognitive behaviour > RDF50102 Cognitive sciences > RDF501 Psychology and cognitive sciences > RDF50 Social sciences	1 (0.1)
RDF3030215 Sex and gender-based analysis > RDF30302 Public and population health > RDF303 Health sciences > RDF30 Medical, health and life sciences	1 (0.1)
RDF3020214 Clinical genetics (except cancer genetics) > RDF30202 Clinical sciences > RDF302 Clinical medicine > RDF30 Medical, health and life sciences	1 (0.1)
RDF1051001 Indigenous peoples environmental knowledge > RDF10510 Natural environment sciences > RDF105 Earth and related environmental sciences > RDF10 Natural sciences	1 (0.1)
RDF3010407 Sensory systems, auditory > RDF30104 Neurosciences, medical and physiological and health aspects > RDF301 Basic medicine and life sciences > RDF30 Medical, health and life sciences	1 (0.1)
RDF5049905 Social oppression and marginalization > RDF50499 Other studies in human society > RDF504 Sociology and related studies > RDF50 Social sciences	1 (0.1)
RDF3030221 Nutritional epidemiology > RDF30302 Public and population health > RDF303 Health sciences > RDF30 Medical, health and life sciences	1 (0.1)
RDF5020299 Industrial relations and work relations, n.e.c. > RDF50202 Industrial relations and work relations > RDF502 Economics and business administration > RDF50 Social sciences	1 (0.1)
RDF3030208 Aging process > RDF30302 Public and population health > RDF303 Health sciences > RDF30 Medical, health and life sciences	1 (0.1)
RDF1060609 Genomics > RDF10606 Genetics > RDF106 Biological sciences > RDF10 Natural sciences	1 (0.1)
RDF3030604 Clinical nursing, tertiary (rehabilitative care) > RDF30306 Nursing > RDF303 Health sciences > RDF30 Medical, health and life sciences	1 (0.1)
RDF3030105 Public health nutrition policy > RDF30301 Human nutrition and dietetics > RDF303 Health sciences > RDF30 Medical, health and life sciences	1 (0.1)
RDF2080105 Wastewater treatment (including water treatment processes) > RDF20801 Environmental engineering > RDF208 Environmental engineering and related engineering > RDF20-21 Engineering and technology	1 (0.1)
RDF3030106 Human nutrition and metabolism > RDF30301 Human nutrition and dietetics > RDF303 Health sciences > RDF30 Medical, health and life sciences	1 (0.1)
RDF5099902 Disability studies > RDF50999 Other social sciences, n.e.c. > RDF509 Other social sciences > RDF50 Social sciences	1 (0.1)
RDF5010123 Personality psychology > RDF50101 Psychology, social and behavioural aspects > RDF501 Psychology and cognitive sciences > RDF50 Social sciences	1 (0.1)
RDF3030301 Kinesiology > RDF30303 Rehabilitation medicine > RDF303 Health sciences > RDF30 Medical, health and life sciences	1 (0.1)

RDF1051003 Environmental impact assessment > RDF10510 Natural environment sciences > RDF105 Earth and related environmental sciences > RDF10 Natural sciences	1 (0.1)
RDF3010108 Adaptive immunity > RDF30101 Immunology > RDF301 Basic medicine and life sciences > RDF30 Medical, health and life sciences	1 (0.1)
RDF1050108 Climate change impacts and adaptation > RDF10501 Atmospheric sciences > RDF105 Earth and related environmental sciences > RDF10 Natural sciences	1 (0.1)
RDF3030303 Occupational therapy > RDF30303 Rehabilitation medicine > RDF303 Health sciences > RDF30 Medical, health and life sciences	1 (0.1)
RDF3010702 Musculoskeletal biology and physiology > RDF30107 Musculoskeletal health and human movement > RDF301 Basic medicine and life sciences > RDF30 Medical, health and life sciences	1 (0.1)
RDF1020110 Data mining > RDF10201 Artificial intelligence (AI) > RDF102 Computer and information sciences > RDF10 Natural sciences	1 (0.1)
RDF1061102 Cellular neuroscience > RDF10611 Neurosciences, biological and chemical aspects > RDF106 Biological sciences > RDF10 Natural sciences	1 (0.1)
RDF3030517 Internal medicine > RDF30305 Care > RDF303 Health sciences > RDF30 Medical, health and life sciences	1 (0.1)
RDF3010303 Medical parasitology > RDF30103 Medical microbiology > RDF301 Basic medicine and life sciences > RDF30 Medical, health and life sciences	1 (0.1)
RDF3030699 Nursing, n.e.c. > RDF30306 Nursing > RDF303 Health sciences > RDF30 Medical, health and life sciences	1 (0.1)
RDF1060102 Enzymes (including kinetics and mechanisms, and biocatalyst) > RDF10601 Biochemistry > RDF106 Biological sciences > RDF10 Natural sciences	1 (0.1)
RDF1060202 Cell metabolism > RDF10602 Cell biology > RDF106 Biological sciences > RDF10 Natural sciences	1 (0.1)
RDF3030499 Health services and systems, n.e.c. > RDF30304 Health services and systems > RDF303 Health sciences > RDF30 Medical, health and life sciences	1 (0.1)
RDF5080304 Technical writing > RDF50803 Journalism and professional writing > RDF508 Media and communications > RDF50 Social sciences	1 (0.1)
RDF2030403 Renewable energy systems (except smart systems engineering) > RDF20304 Electrical energy systems > RDF203 Electrical engineering, computer engineering, and information engineering > RDF20-21 Engineering and technology	1 (0.1)
RDF3020233 Clinical toxicology > RDF30202 Clinical sciences > RDF302 Clinical medicine > RDF30 Medical, health and life sciences	1 (0.1)
RDF3010308 Human microbiota > RDF30103 Medical microbiology > RDF301 Basic medicine and life sciences > RDF30 Medical, health and life sciences	1 (0.1)
RDF2110112 Nanotoxicology, health and safety > RDF21101 Nano-technology > RDF211 Nano-technology > RDF20-21 Engineering and technology	1 (0.1)

RDF3010399 Medical microbiology, n.e.c. > RDF30103 Medical microbiology > RDF301 Basic medicine and life sciences > RDF30 Medical, health and life sciences	1 (0.1)
RDF3010103 Autoimmunity > RDF30101 Immunology > RDF301 Basic medicine and life sciences > RDF30 Medical, health and life sciences	1 (0.1)
RDF2070103 Biomedical instrumentation (including diagnostics) > RDF20701 Medical and biomedical engineering > RDF207 Medical and biomedical engineering > RDF20-21 Engineering and technology	1 (0.1)
RDF5010205 Cognitive understanding > RDF50102 Cognitive sciences > RDF501 Psychology and cognitive sciences > RDF50 Social sciences	1 (0.1)
RDF3020230 Clinical sexology > RDF30202 Clinical sciences > RDF302 Clinical medicine > RDF30 Medical, health and life sciences	1 (0.1)
RDF3030222 One Health approach > RDF30302 Public and population health > RDF303 Health sciences > RDF30 Medical, health and life sciences	1 (0.1)
RDF3020105 Respiratory sciences > RDF30201 Cardiorespiratory medicine and hematology > RDF302 Clinical medicine > RDF30 Medical, health and life sciences	1 (0.1)
RDF3020106 Respiratory system pathologies and clinical interventions > RDF30201 Cardiorespiratory medicine and hematology > RDF302 Clinical medicine > RDF30 Medical, health and life sciences	1 (0.1)
RDF5030202 Curriculum and pedagogy theory and development > RDF50302 Curriculum, pedagogy and didactics > RDF503 Education > RDF50 Social sciences	1 (0.1)
RDF3030510 Hospital and specialist care > RDF30305 Care > RDF303 Health sciences > RDF30 Medical, health and life sciences	1 (0.1)
RDF5030308 Gender, Sexuality and education > RDF50303 Specialized studies in education > RDF503 Education > RDF50 Social sciences	1 (0.1)
RDF3020201 Anesthesiology > RDF30202 Clinical sciences > RDF302 Clinical medicine > RDF30 Medical, health and life sciences	1 (0.1)
RDF5040111 Urban sociology and community studies > RDF50401 Sociology > RDF504 Sociology and related studies > RDF50 Social sciences	1 (0.1)
RDF3020202 Clinical microbiology > RDF30202 Clinical sciences > RDF302 Clinical medicine > RDF30 Medical, health and life sciences	1 (0.1)
RDF3020203 Dermatology > RDF30202 Clinical sciences > RDF302 Clinical medicine > RDF30 Medical, health and life sciences	1 (0.1)
RDF3010423 Applied behavioural neurology and neuropsychiatry > RDF30104 Neurosciences, medical and physiological and health aspects > RDF301 Basic medicine and life sciences > RDF30 Medical, health and life sciences	1 (0.1)
RDF3030307 Sports medicine > RDF30303 Rehabilitation medicine > RDF303 Health sciences > RDF30 Medical, health and life sciences	1 (0.1)
RDF5090304 Mortality > RDF50903 Demography > RDF509 Other social sciences > RDF50 Social sciences	1 (0.1)
RDF3020503 Pediatrics > RDF30205 Pediatrics and reproductive medicine > RDF302 Clinical medicine > RDF30 Medical, health and life sciences	1 (0.1)

RDF5099901 Sexology > RDF50999 Other social sciences, n.e.c. > RDF509 Other social sciences > RDF50 Social sciences	1 (0.1)
RDF3020504 Human reproduction and development sciences > RDF30205 Pediatrics and reproductive medicine > RDF302 Clinical medicine > RDF30 Medical, health and life sciences	1 (0.1)
RDF6010303 Religion and spirituality of Indigenous peoples > RDF60103 Religion and religious studies > RDF601 History, archaeology and related studies > RDF60 Humanities and the arts	1 (0.1)
RDF3020505 Neonatology > RDF30205 Pediatrics and reproductive medicine > RDF302 Clinical medicine > RDF30 Medical, health and life sciences	1 (0.1)
RDF3020506 Embryology > RDF30205 Pediatrics and reproductive medicine > RDF302 Clinical medicine > RDF30 Medical, health and life sciences	1 (0.1)
