# Supplementary material for: Characterizing Canadian funded partnered health research projects between 2011 and 2019: a retrospective analysis
Source: Health Res Policy Syst. 2023 Sep 8;21:92. doi: 10.1186/s12961-023-01046-x (PMC10492355; doi:10.1186/s12961-023-01046-x)
Supplement: Supplementary file 3 — Additional file 3: Appendix 3. Type of Research codes (N = 1143). [file 12961_2023_1046_MOESM3_ESM.pdf]

Appendix 3: Type of Research codes (N=1143)

**Code (Class > Group)**	**# (%)**
Policy, ethics and research governance > Health and social care services research	479 (41.9)
Organisation and delivery of services > Health and social care services research	215 (18.8)
Individual care needs > Management of diseases and conditions	68 (5.9)
Management and decision making > Management of diseases and conditions	61 (5.3)
Research design and methodologies > Health and social care services research	31 (2.7)
Resources and infrastructure (health services) > Health and social care services research	27 (2.4)
Psychological, social and economic factors > Aetiology	22 (1.9)
Primary prevention interventions to modify behaviours or promote wellbeing > Prevention of disease and conditions, and promotion of wellbeing	22 (1.9)
Cellular and gene therapies > Development of treatments and therapeutic interventions	19 (1.7)
Psychological and socioeconomic processes > Underpinning research	18 (1.6)
Biological and endogenous factors >Aetiology	17 (1.5)
Discovery and preclinical testing of markers and technologies > Detection, screening and diagnosis	15 (1.3)
Psychological and behavioural > Evaluation of treatments and therapeutic interventions	12 (1)
Evaluation of markers and technologies > Detection, screening and diagnosis	12 (1)
End of life care >Management of diseases and conditions	11 (1)
Health and welfare economics > Health and social care services research	10 (0.9)
Physical > Evaluation of treatments and therapeutic interventions	10 (0.9)
Pharmaceuticals > Development of treatments and therapeutic interventions	8 (0.7)
Factors relating to physical environment > Aetiology	7 (0.6)
Surveillance and distribution > Aetiology	7 (0.6)
Resources and infrastructure (disease management) >Management of diseases and conditions	6 (0.5)
Pharmaceuticals > Evaluation of treatments and therapeutic interventions	6 (0.5)
Vaccines > Prevention of disease and conditions, and promotion of wellbeing	6 (0.5)
Surgery > Development of treatments and therapeutic interventions	5 (0.4)
Radiotherapy and other non-invasive therapies > Development of treatments and therapeutic interventions	5 (0.4)

Medical devices > Evaluation of treatments and therapeutic interventions	5 (0.4)
Population screening > Detection, screening and diagnosis	5 (0.4)
Influences and impact > Detection, screening and diagnosis	5 (0.4)
Interventions to alter physical and biological environmental risks > Prevention of disease and conditions, and promotion of wellbeing	5 (0.4)
Complementary > Evaluation of treatments and therapeutic interventions	4 (0.3)
Psychological and behavioural > Development of treatments and therapeutic interventions	3 (0.3)
Physical > Development of treatments and therapeutic interventions	3 (0.3)
Surgery > Evaluation of treatments and therapeutic interventions	2 (0.2)
Cellular and gene therapies > Evaluation of treatments and therapeutic interventions	2 (0.2)
Nutrition and chemoprevention > Prevention of disease and conditions, and promotion of wellbeing	2 (0.2)
Normal biological development and functioning > Underpinning research	2 (0.2)
Resources and infrastructure (evaluation of treatments) > Evaluation of treatments and therapeutic interventions	1 (0.1)
Resources and infrastructure (aetiology) > Aetiology	1 (0.1)
Resources and infrastructure (development of treatments) > Development of treatments and therapeutic interventions	1 (0.1)
Resources and infrastructure (prevention) > Prevention of disease and conditions, and promotion of wellbeing	1 (0.1)
Chemical and physical sciences > Underpinning research	1 (0.1)
Resources and infrastructure (underpinning) > Underpinning research	1 (0.1)
