# Supplementary material for: Characterizing Canadian funded partnered health research projects between 2011 and 2019: a retrospective analysis
Source: Health Res Policy Syst. 2023 Sep 8;21:92. doi: 10.1186/s12961-023-01046-x (PMC10492355; doi:10.1186/s12961-023-01046-x)
Supplement: Supplementary file 4 — Additional file 4: Appendix 4. Top five Field of Research codes by funding year block. [file 12961_2023_1046_MOESM4_ESM.pdf]

Appendix 4: Top five Field of Research codes by funding year block

Funding year block	Rank	Subclass	Class	Group	Division	Number of Projects (%)
2011-13 (n=406)	1	Infectious diseases	Clinical sciences	Clinical medicine	Medical, health and life sciences	19 (4.7)
	1	Health care safety and quality improvement	Health services and systems	Health sciences	Medical, health and life sciences	19 (4.7)
	3	Health care effectiveness and outcomes	Health services and systems	Health sciences	Medical, health and life sciences	15 (3.7)
	4	Emergency care and critical care	Care	Health sciences	Medical, health and life sciences	12 (2.9)
	5	Cardiology and circulatory sciences (including cardiovascular disease)	Cardiorespiratory medicine and hematology	Clinical medicine	Medical, health and life sciences	11 (2.7)
2014-16 (n=407)	1	Infectious diseases	Clinical sciences	Clinical medicine	Medical, health and life sciences	20 (4.9)
	2	Health care safety and quality improvement	Health services and systems	Health sciences	Medical, health and life sciences	19 (4.7)
	3	Emergency care and critical care	Care	Health sciences	Medical, health and life sciences	15 (3.7)
	4	Mental health and wellbeing	Psychology, social and behavioural aspects	Psychology and cognitive sciences	Social sciences	13 (3.2)
	5	Health care effectiveness and outcomes	Health services and systems	Health sciences	Medical, health and life sciences	12 (2.9)
2017-19 (n=339)	1	Infectious diseases	Clinical sciences	Clinical medicine	Medical, health and life sciences	18 (5.3)
	2	Health care safety and quality improvement	Health services and systems	Health sciences	Medical, health and life sciences	15 (4.4)
	3	Health equity	Public and population health	Health sciences	Medical, health and life sciences	13 (3.8)

	4	Addiction rehabilitation	Rehabilitation medicine	Health sciences	Medical, health and life sciences	13 (3.8)
	5	Mental health and wellbeing	Psychology, social and behavioural aspects	Psychology and cognitive sciences	Social sciences	10 (2.9)
