# Supplementary material for: Characterizing Canadian funded partnered health research projects between 2011 and 2019: a retrospective analysis
Source: Health Res Policy Syst. 2023 Sep 8;21:92. doi: 10.1186/s12961-023-01046-x (PMC10492355; doi:10.1186/s12961-023-01046-x)
Supplement: Supplementary file 5 — Additional file 5: Appendix 5. Top five Type of Research codes by funding year block. [file 12961_2023_1046_MOESM5_ESM.pdf]

Appendix 5: Top five Type of Research codes by funding year block

Funding year block	Rank	Type of Research Code	Research activity group	Number of Projects (%)
2011-13 (n=403)	1	Policy, ethics and research governance	Health and social care services research	198 (49.1)
	2	Organisation and delivery of services	Health and social care services research	56 (13.9)
	3	Management and decision making	Management of diseases and conditions	35 (8.7)
	4	Individual care needs	Management of diseases and conditions	23 (5.7)
	5	Research design and methodologies	Health and social care services research	10 (2.5)
2014-16 (n=402)	1	Policy, ethics and research governance	Health and social care services research	159 (39.5)
	2	Organisation and delivery of services	Health and social care services research	75 (18.6)
	3	Individual care needs	Management of diseases and conditions	27 (6.7)
	4	Resources and infrastructure (health services)	Health and social care services research	14 (3.5)
	5	Management and decision making	Management of diseases and conditions	14 (3.5)
2017-19 (n=338)	1	Policy, ethics and research governance	Health and social care services research	122 (36.1)
	2	Organisation and delivery of services	Health and social care services research	84 (24.9)
	3	Individual care needs	Management of diseases and conditions	18 (5.3)
	4	Management and decision making	Management of diseases and conditions	12 (3.6)
	5	Research design and methodologies	Health and social care services research	11 (3.3)
