# Supplementary material for: Characterizing Canadian funded partnered health research projects between 2011 and 2019: a retrospective analysis
Source: Health Res Policy Syst. 2023 Sep 8;21:92. doi: 10.1186/s12961-023-01046-x (PMC10492355; doi:10.1186/s12961-023-01046-x)
Supplement: Supplementary file 6 — Additional file 6: Appendix 6. Top five Field of Research codes by partnership type. [file 12961_2023_1046_MOESM6_ESM.pdf]

Appendix 6: Top five Field of Research codes by partnership type

Partnership type	Rank	Number of projects (%)	Subclass	Class	Group	Division
Required N=925	1	45 (4.8)	Health care safety and quality improvement	Health services and systems	Health sciences	Medical, health and life sciences
	2	43 (4.6)	Infectious diseases	Clinical sciences	Clinical medicine	Medical, health and life sciences
	3	29 (3.1)	Health care effectiveness and outcomes	Health services and systems	Health sciences	Medical, health and life sciences
	4	27 (2.9)	Mental health and wellbeing	Psychology, social and behavioural aspects	Psychology and cognitive sciences	Social sciences
	5	24 (2.6)	Cardiology and circulatory sciences (including cardiovascular disease)	Cardiorespiratory medicine and hematology	Clinical medicine	Medical, health and life sciences
Optional N=228	1	14 (6.1)	Infectious diseases	Clinical sciences	Clinical medicine	Medical, health and life sciences
	2	12 (5.3)	Emergency care and critical care	Care	Health sciences	Medical, health and life sciences
	3	9 (3.9)	Coordinated and integrated care	Care	Health sciences	Medical, health and life sciences
	4	8 (3.5)	Primary health care	Care	Health sciences	Medical, health and life sciences
	5	8 (3.5)	Health equity	Public and population health	Health sciences	Medical, health and life sciences
