# Supplementary material for: Characterizing Canadian funded partnered health research projects between 2011 and 2019: a retrospective analysis
Source: Health Res Policy Syst. 2023 Sep 8;21:92. doi: 10.1186/s12961-023-01046-x (PMC10492355; doi:10.1186/s12961-023-01046-x)
Supplement: Supplementary file 7 — Additional file 7: Appendix 7. Top five Type of Research codes by partnership type. [file 12961_2023_1046_MOESM7_ESM.pdf]

Appendix 7: Top five Type of Research codes by partnership type

Partnership type	Rank	Number of projects (%)	Type of research code	Research activity group
Required N=925	1	420 (45.4)	Policy, ethics and research governance	Health and social care services research
	2	165 (17.8)	Organisation and delivery of services	Health and social care services research
	3	52 (5.6)	Individual care needs	Management of diseases and conditions
	4	42 (4.5)	Management and decision making	Management of diseases and conditions
	5	20 (2.2)	Resources and infrastructure (health services)	Health and social care services research
Optional N=228	1	59 (25.8)	Policy, ethics and research governance	Health and social care services research
	2	50 (21.9)	Organisation and delivery of services	Health and social care services research
	3	19 (8.3)	Management and decision making	Management of diseases and conditions
	4	16 (7)	Individual care needs	Management of diseases and conditions
	5	13 (5.7)	Research design and methodologies	Health and social care services research
