# Supplementary material for: Characterizing Canadian funded partnered health research projects between 2011 and 2019: a retrospective analysis
Source: Health Res Policy Syst. 2023 Sep 8;21:92. doi: 10.1186/s12961-023-01046-x (PMC10492355; doi:10.1186/s12961-023-01046-x)
Supplement: Supplementary file 8 — Additional file 8: Appendix 8. Top five Field of Research codes over time by funder type. [file 12961_2023_1046_MOESM8_ESM.pdf]

Appendix 8: Top five Field of Research codes over time by funder type

Funder type	Funding year block	Rank	Number of projects (%)	Subclass	Class	Group	Division
CIHR (851)	2011-13 N=349	1	19 (5.4)	Infectious diseases	Clinical sciences	Clinical medicine	Medical, health and life sciences
		2	16 (4.6)	Health care safety and quality improvement	Health services and systems	Health sciences	Medical, health and life sciences
		3	12 (3.4)	Health care effectiveness and outcomes	Health services and systems	Health sciences	Medical, health and life sciences
		4	10 (2.9)	Cardiology and circulatory sciences (including cardiovascular disease)	Cardiorespiratory medicine and hematology	Clinical medicine	Medical, health and life sciences
		5	9 (2.6)	Primary health care	Care	Health sciences	Medical, health and life sciences
	2014-16 N=292	1	17 (5.8)	Infectious diseases	Clinical sciences	Clinical medicine	Medical, health and life sciences
		2	13 (4.4)	Health care safety and quality improvement	Health services and systems	Health sciences	Medical, health and life sciences
		3	12 (4.1)	Emergency care and critical care	Care	Health sciences	Medical, health and life sciences
		4	10 (3.4)	Mental health and wellbeing	Psychology, social and behavioural aspects	Psychology and cognitive sciences	Social sciences
		5	8 (2.7)	Aged health care services (except nursing)	Care	Health sciences	Medical, health and life sciences
	2017-19 N=210	1	14 (6.6)	Infectious diseases	Clinical sciences	Clinical medicine	Medical, health and life sciences
		2	9 (4.3)	Health care safety and quality improvement	Health services and systems	Health sciences	Medical, health and life sciences
		3	9 (4.3)	Health equity	Public and population health	Health sciences	Medical, health and life sciences

		4	8 (3.8)	Maternal and newborn care			
		5	8 (3.8)	Mental health and wellbeing	Psychology, social and behavioural aspects	Psychology and cognitive sciences	Social sciences
Provincial N=351	2011-13 N=57	1	3 (5.3)	Health care safety and quality improvement	Health services and systems	Health sciences	Medical, health and life sciences
		2	3 (5.3)	Health care effectiveness and outcomes	Health services and systems	Health sciences	Medical, health and life sciences
		3	3 (5.3)	Cancer diagnosis	Cancer	Basic medicine and life sciences	Basic medicine and life sciences
		4	3 (5.3)	Emergency care and critical care	Care	Health sciences	Medical, health and life sciences
		5	2 (3.5)	Solid cancer tumours	Cancer	Basic medicine and life sciences	Basic medicine and life sciences
	2014-16 N=116	1	6 (5.2)	Health care safety and quality improvement	Health services and systems	Health sciences	Medical, health and life sciences
		2	5 (4.3)	Nephrology	Clinical sciences	Clinical medicine	Medical, health and life sciences
		3	5 (4.3)	Health care effectiveness and outcomes	Health services and systems	Health sciences	Medical, health and life sciences
		4	4 (3.4)	Cancer progression and metastasis	Cancer	Basic medicine and life sciences	Basic medicine and life sciences
		5	4 (3.4)	Palliation and end-of-life care	Care	Health sciences	Basic medicine and life sciences
	2017-19 N=129	1	6 (4.7)	Health care safety and quality improvement	Health services and systems	Health sciences	Medical, health and life sciences
		2	5 (3.9)	Gender and health relationship	Public and population health	Health sciences	Medical, health and life sciences
		3	5 (3.9)	Addiction rehabilitation	Rehabilitation medicine	Health sciences	Medical, health and life sciences

		4	4 (3.1)	Infectious diseases	Clinical sciences	Clinical medicine	Medical, health and life sciences
		5	4 (3.1)	Health care access, privilege or marginalization	Care	Health sciences	Basic medicine and life sciences
