# Supplementary material for: Characterizing Canadian funded partnered health research projects between 2011 and 2019: a retrospective analysis
Source: Health Res Policy Syst. 2023 Sep 8;21:92. doi: 10.1186/s12961-023-01046-x (PMC10492355; doi:10.1186/s12961-023-01046-x)
Supplement: Supplementary file 9 — Additional file 9: Appendix 9. Top five Type of Research codes over time by funder type. [file 12961_2023_1046_MOESM9_ESM.pdf]

Appendix 9: Top five Type of Research codes over time by funder type

Partnership type	Funding year block	Rank	Number of projects (%)	Type of research code	Research activity group
CIHR N=925	2011-13 N=349	1	195 (55.9)	Policy, ethics and research governance	Health and social care services research
		2	43 (12.3)	Organisation and delivery of services	Health and social care services research
		3	33 (9.5)	Management and decision making	Management of diseases and conditions
		4	15 4.3)	Individual care needs	Management of diseases and conditions
		5	10 2.9)	Research design and methodologies	Health and social care services research
	2014-16 N=292	1	147 (50.3)	Policy, ethics and research governance	Health and social care services research
		2	49 (16.8)	Organisation and delivery of services	Health and social care services research
		3	14 (4.8)	Individual care needs	Management of diseases and conditions
		4	10 (3.4)	Research design and methodologies	Health and social care services research
		5	9 (3.1)	Management and decision making	Management of diseases and conditions
	2017-19 N=210	1	59 (28.1)	Policy, ethics and research governance	Health and social care services research
		2	57 (27.1)	Organisation and delivery of services	Health and social care services research
		3	13 (6.1)	Individual care needs	Management of diseases and conditions
		4	11 (5.2)	Research design and methodologies	Health and social care services research
		5	9 (4.3)	Management and decision making	Management of diseases and conditions
Provincial N=351	2011-13 N=57	1	13 (22.8)	Organisation and delivery of services	Health and social care services research
		2	8 (14)	Individual care needs	Management of diseases and conditions
		3	5 (8.8)	Cellular and gene therapies	Development of treatments and therapeutic interventions
		4	3 (5.3)	Surgery	Development of treatments and therapeutic interventions
		5	3 (5.3)	Policy, ethics and research governance	Health and social care services research
	2014-16 N=116	1	26 (22.4)	Organisation and delivery of services	Health and social care services research
		2	13 (11.2)	Individual care needs	Management of diseases and conditions
		3	12 (10.3)	Policy, ethics and research governance	Health and social care services research
		4	9 (7.7)	Cellular and gene therapies	Development of treatments and therapeutic interventions

		5	5 (4.3)	Management and decision making	Management of diseases and conditions
	2017-19 N=129	1	63 (48.8)	Policy, ethics and research governance	Health and social care services research
		2	27 (20.9)	Organisation and delivery of services	Health and social care services research
		3	5 (3.9)	Individual care needs	Management of diseases and conditions
		4	4 (3.1)	Discovery and preclinical testing of markers and technologies	Detection, screening and diagnosis
		5	3 (2.3)	Primary prevention interventions to modify behaviours or promote wellbeing	Prevention of disease and conditions, and promotion of wellbeing
